# Exploring the Control in Antibacterial Activity of Silver Triangular Nanoplates by Surface Coating Modulation

**DOI:** 10.3389/fchem.2018.00677

**Published:** 2019-02-05

**Authors:** Jamila Djafari, Carlos Fernández-Lodeiro, Adrián Fernández-Lodeiro, Vanessa Silva, Patrícia Poeta, Gilberto Igrejas, Carlos Lodeiro, José Luis Capelo, Javier Fernández-Lodeiro

**Affiliations:** ^1^BIOSCOPE Group, LAQV@REQUIMTE, Chemistry Department, Faculty of Science and Technology, NOVA University Lisbon, Caparica, Portugal; ^2^PROTEOMASS Scientific Society, Rua dos Inventores, Madam Parque, Caparica, Portugal; ^3^Associated Laboratory for Green Chemistry (LAQV-REQUIMTE), University NOVA of Lisbon, Caparica, Portugal; ^4^Department of Genetics and Biotechnology, University of Trás-os-Montes and Alto Douro, Vila Real, Portugal; ^5^Functional Genomics and Proteomics Unit, University of Trás-os-Montes and Alto Douro, Vila Real, Portugal; ^6^Veterinary Science Department, University of Trás-os-Montes and Alto Douro, Vila Real, Portugal

**Keywords:** silver triangular nanoplates, silica coating, succinic anhydride, APTMS, antibacterial properties

## Abstract

In the present work, the synthesis and characterization of silver triangular nanoplates (AgNTs) and their silica coating composites are reported. Engineering control on the surface coating has demonstrated the possibility to modulate the antibacterial effect. Several AgNT-coated nanomaterials, such as PVP (Polyvinylpyrrolidone) and MHA (16-mercaptohexadecanoic acid) as a stable organic coating system as well as uniform silica coating (≈5 nm) of AgNTs, have been prepared and fully characterized. The antibacterial properties of the systems reported, organic (MHA) and inorganic (amine and carboxylic terminated SiO_2_) coating nanocomposites, have been tested on Gram-positive and Gram-negative bacteria strains. We observed that the AgNTs' organic coating improved antimicrobial properties when compared to other spherical silver colloids found in the literature. We have also found that thick inorganic silica coating decreases the antimicrobial effect, but does not cancel it. In addition, the effect of surface charge in AgNTs@Si seems to play a crucial role toward *S. aureus* ATCC 25923 bacteria, obtaining MIC/MBC values compared to the AgNTs with an organic coating.

## Introduction

Silver nanoparticles (AgNPs) have attracted much attention as a result of their particular optoelectronic (Kelly et al., 2003; Wei, 2011), catalytic (Jiang et al., 2005; Köhler et al., 2008), or antibacterial properties (Morones et al., 2005; Rai et al., 2009).

Engineering modifications of AgNPs' size and shape represent a fascinating synthetic challenge that allow modification of the final nanomaterial's properties. These structural modifications at the nanoscale level strongly affect the macroscopic properties of the silver colloidal solutions. For instance, the intense colors of silver colloids are the result of different electron oscillation modes that arise when an electromagnetic field, in the visible range, is coupled to the collective oscillations of conduction electrons (Kelly et al., 2003). The optical properties can be significantly modified by adjusting the size and/or the shape of the NPs, allowing a spectral tuning that ranges from the visible to the near-IR region. This is particularly true for anisotropic structures such as nanoprisms or nanoplates, among others (Pastoriza-Santos and Liz-Marzán, 2008; Millstone et al., 2009).

Similarly to optoelectronic properties, the chemical behavior of silver colloids such as catalytic (Kundu et al., 2017) or antibacterial properties (Sadeghi et al., 2012) are also much affected by these structural changes. In this regard, many researchers have stated that AgNPs' chemical performance seems to be related with the different reactivity of the atoms located at the intersections, or in the corners of these nanostructures (Le Beulze et al., 2014; Kundu et al., 2017).

Furthermore, the antibacterial effect is one of the most explored applications owing to its excellent effect against a broad spectrum of bacteriological organisms. During the previous decades, the scientific community has debated over the different mechanisms in which AgNPs exert their toxicity toward bacteria and other microorganisms. It has been proved in numerous studies the crucial role of silver ions (Ag^+^) release in the mechanism of antibacterial action of AgNPs (Xiu et al., 2012). In this vein, it has been suggested that the morphology of the AgNPs also affects the antimicrobial activity, as an essential indirect factor that mainly influences the release of Ag^+^ (Feng et al., 2000; Xiu et al., 2012).

In this respect, several studies have shown that silver nanoprisms (AgNTs) have more bactericidal action than nanorods or nanospheres, demonstrating that the nanomaterial's shape strongly influences the bactericidal effect of silver nanoparticles (Xue et al., 2007; Van Dong et al., 2012; Pal et al., 2015). Indeed, the presence of high atomic density facets in nanoprim structures such as {111} (like triangular or decahedral shape), induces the increase of nanoparticle antibacterial activity and seems to be important in the direct interaction within the bacteria surface (Morones et al., 2005; Sadeghi et al., 2012). Furthermore, AgNTs exhibit high surface energy, mainly located at their tips and edges, where silver atoms can be readily oxidized, resulting in either truncation of prism tips or their complete dissolution (Pastoriza-Santos and Liz-Marzán, 2008; Millstone et al., 2009). This significant drawback can significantly limit their physicochemical properties' advantages and therefore could reduce the antibacterial application of these nanostructures. Different coating methods have been developed in order to avoid this disadvantage, minimizing the effect, and enabling the manipulation of this material as a building block in future applications.

Many studies around spherical AgNPs have proved that whether there is organic (Xiu et al., 2012; Yang et al., 2012; Abbaszadegan et al., 2015) or inorganic (usually mesoporous silica) coating (Liong et al., 2009; Le et al., 2010; Nuti et al., 2018) has an essential influence on AgNPs' antibacterial effect. In this regard, it has been confirmed that the Ag^+^ release could be controlled, and as an important consequence, the environmental impacts could be strongly mitigated.

In contrast, the case of AgNTs has been much less investigated. A. Yu et al. provided a significant advance about AgNT stabilization using different alkanethiols (Jiang et al., 2007). These authors report that the Ag-S interactions considerably delay the dissolution of AgNT structures. These important chemical observations were later exploited by Xue et al. (2007), in an elegant work in which the authors demonstrated the perfect AgNT silica coating.

Additionally, it has been demonstrated that amorphous silica coating over AgNPs presents porosity, allowing the diffusion of ions that can oxidize the silver core (Mulvaney et al., 1997). The porosity of the amorphous silica has already been proved in studies by different groups (Lecloux et al., 1986; van Blaaderen and Vrij, 1993). Nevertheless, unfortunately, according to the best of our knowledge, the antibacterial properties of AgNTs subjected to alkanethiol or silica coating have not yet been explored.

The goal of the present work is, therefore, to investigate the influences of surface coating of well-defined AgNTs (organic and inorganic) on their optical properties as well as the effects of antibacterial activity against *E. coli* and *S. aureus*. The effect of the surface charge and terminal functional groups (NH_2_ or COOH) on AgNTs@SiO_2_ was also investigated.

## Materials and Methods

### Materials

Silver nitrate 99% (AgNO_3_), sodium borohydride 99% (NaBH_4_), Sodium citrate tribasic dihydrate 99%, hydrogen peroxide 30% (H_2_O_2_), polyvinylpyrrolidone (PVP-29K), 16-mercaptohexadecanoic acid 90% (MHA), dimethylamine (DMA) 40% in water, tetraethylorthosilicate (TEOS), (3-Aminopropyl)trimethoxysilane 97% (APTMS), Succinic anhydride 99% (SA), and anhydrous Tetrahydrofuran (a-THF) were obtained from Sigma-Aldrich, and used without previous purification. Anhydrous Ethanol (a-EtOH) was purchased from Carlo Elba. Water was used at Milli-Q grade by Millipore (MQ).

### Methods

#### Synthesis of AgNTs in Water (AgNTs@PVP) and MHA Stabilization (AgNTs@MHA)

The synthesis was carried out in a total volume of 50 mL of MQ water at 30°C, under ambient atmosphere and laboratory light. Over an aqueous solution of AgNO_3_ (final concentration of 0.2 mM), under vigorous stirring, trisodium citrate (150 mM, 1 mL), PVP 29K (135 mg/mL, 3 mL), and hydrogen peroxide (30 wt%, 240 μL) aqueous solutions were added. Afterward, a freshly prepared aqueous solution of NaBH_4_ (final concentration of 1.6 mM) was rapidly added. The solution then immediately turned clear yellow. After 10 min, the colloid solution changed to intense yellow, showing the formation of spherical silver nanoparticles, and then the color solution turned to orange, red, purple, and finally blue. The silver nanoplates were centrifuged at 10,000 rpm during 30 min and re-dispersed in ethanol.

The coating of AgNTs@PVP with 16-MHA was carried out by quickly adding an ethanolic solution of MHA (final concentration of 60 μM) on the AgNTs@PVP in EtOH under vigorous stirring and darkness. After 15 min, the colloid was centrifugated at 11,000 rpm and re-suspended in ethanol.

#### Silica Coating of AgNTs@MHA to Produce AgNTs@Si-OH

An ethanolic solution of TEOS (final concentrations explored between 0.9 and 0.4 mM) was added to the colloid AgNTs@MHA in EtOH. Then, an aqueous DMA solution was rapidly injected into the mixture (final concentrations explored between 0.6 and 0.4 M). The solution was left stirring for 180 or 90 min. at ambient temperature in the dark. The AgNTs@Si-OH were centrifuged several times and washed in ethanol and ultra-pure water. Then, the purified colloid was resuspended in ethanol.

#### Amine Derivatization of AgNTs@Si-OH to Produce AgNTs@Si-NH_2_

The AgNTs@Si-OH colloid was coated with amine silane to convert the AgNTs' surface with amine function. Briefly, under stirring, APTMS in ethanol solution (final concentration of 33.7 μM) was added to an ethanolic solution of AgNTs@Si-OH. Then, 1.32% (of total volume reaction) of Milli-Q water was added as a catalyzer agent (Bruce and Sen, 2005). The reaction was left under stirring overnight. The resulting solution was purified by repeated centrifugation using ethanol and Milli-Q water.

#### Carboxylic Acid Derivatization of AgNTs@Si-NH_2_ to Produce AgNTs@Si-COOH

The AgNTs@Si-NH_2_ were washed several times in a-THF, and finally suspended in anhydrous THF. A solution of succinic anhydride in a-THF was added drop-wise to the colloid solution AgNTs@Si-NH_2_ until it reached a concentration of 0.52 mM. The reaction was then stirred for 24 h under dark conditions. The resulting AgNTs@Si-COOH were purified several times using THF and then Milli-Q.

#### Characterization Technics

##### UV/Vis spectroscopy analysis

The UV/Vis spectroscopy studies were performed using a JASCO 630 spectrophotometer provided by the PROTEOMASS-BIOSCOPE facility. The spectra were run between 200 and 1,000 nm using a quartz cell (1 cm pathway) under temperature control.

##### Z-Potential analysis

A MALVERN model ZS instrument provided by the PROTEOMASS-BIOSCOPE facility was used to obtain the Z potential values. A “dip” cell was used to measure the Z potential.

##### Transmission electron microscopy (TEM) analysis

Microscopy analyses were performed at the CACTI, University of Vigo (Spain). A JEOL JEM1010 TEM operating at 100 kV was used. All TEM samples were prepared by placing a drop of the sample (5 μL) on a TEM copper grid and then air-dried.

##### Inductively coupled plasma (ICP) analysis

Ag contents in each studied sample were determined in the REQUIMTE-Chemistry Department, FCT-UNL analytical laboratory using an ICP instrument from Horiba Jobin-Yvon (France, model Ultima), equipped with an RF of 40.68 MHz, a 1.00 m Czerny-Turner monochromator (sequential), and an AS500 autosampler.

##### Fourier-transform infrared spectroscopy (FTIR) analysis

A Bruker TENSOR spectrophotometer was used to obtain the FT-IR spectra. FT-IR experiments were performed in a KBr disk, provided by the Chemistry Department, LAQV-REQUIMTE, FCT Facilities. To obtain the KBR discs for analysis, each sample was centrifuged and washed in absolute EtOH several times until finally resuspending each sample in 100μL of anhydrous EtOH. These concentrated solutions were mixed with KBr, and the solids were dried under a vacuum pump for 4 h before the preparation of the disks.

#### Bacterial Strains, Culture Media, and Growth Conditions

The bacterial strains considered in this study were *Escherichia coli* ATCC 29425 and *Staphylococcus aureus* ATCC 25923 (Table 1). Bacterial strains were grown in BHI agar (Oxoid, UK) for 24 h at 37°C.

**Table 1:** Different strains used in the present study.

**Strain**	**Relevant phenotype**	**Reference**
*E. coli* K12 ATCC 29425	Gram-negative	ATCC
*S. aureus* ATCC 25923	Gram-positive	ATCC

#### Preparation of Stock Solutions

Each solution containing AgNTs was diluted to final concentrations of 1, 5, 10, 25, 50, 75, 100, 150, 200, 300, and 500 μg/mL, and tested on both bacteria.

#### Antibacterial Susceptibility Test

The minimum inhibitory concentration (MIC), described as the lowest concentration of nanoparticles that inhibits bacterial growth, was determined by broth-dilution method using a 96-well polystyrene microtiter plate. Luria-Bertani (LB) (Sigma–Aldrich) broth was prepared, and 135 μL was added to each well. Ten microliters of each solution containing different NPs with concentrations ranging from 1 to 500 μg/mL were added to each well, and 10 μL of overnight cultures of the selected bacteria were inoculated into the wells and incubated at 37°C. After 24 h, the absorbance was measured with a microplate spectrophotometer. Positive (inoculated medium) and negative controls (medium supplemented with NPs) were included for all tests. All tests were performed in triplicate.

To determine the minimum bactericidal concentration (MBC), which is characterized by no bacterial growth, 100 μL of the cultures resulting from MIC testing were inoculated onto LB medium plates and incubated at 37°C for 20 h. Control cultures without NPs were included in all experiments.

## Results and Discussion

### Synthesis of Silver Triangular Nanoplates and Alkane-Thiol Functionalization

In the present work, all the experiments have been conceived to determine the antibacterial effects of AgNTs as a function of the surface coating. In the literature, a variety of works that report the synthesis of different triangular nanoplates or nanoprisms with an organic coating to explore their antibacterial properties can be found, but to the best of our knowledge only two works report the controlled silica deposition on AgNTs, and none of them explores the antibacterial properties of the resulting product (Xue et al., 2007; Brandon et al., 2014).

In the first synthetic step, we synthesized AgNTs@PVP using a *non-seed mediated methodology* based on thermal synthesis developed by Métraux and Mirkin (2005) and lately revisited by Yin and coworkers (Zhang et al., 2011; Yu et al., 2014). This photochemical reaction was performed in water with several modifications (see experimental section). We used PVP (29K) as a stabilizer and sodium borohydride as a reducing agent, in the presence of shape directors: citrate ion and hydrogen peroxide.

**Figure 1:**
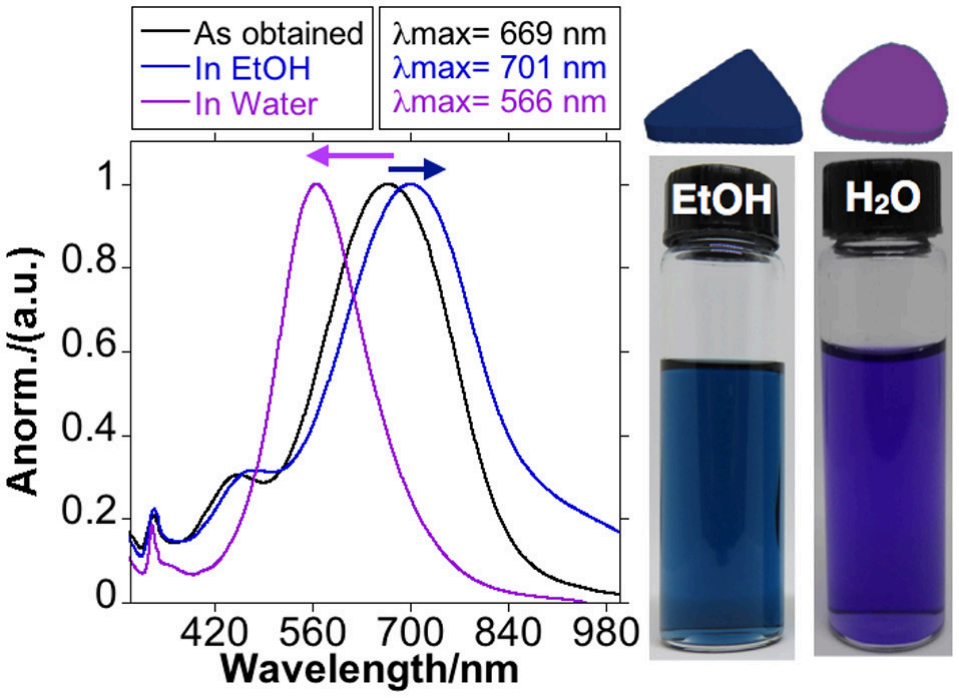
Spectroscopic profile and color of AgNTs@PVP resuspended in EtOH (blue) or Water (purple).

As can be seen in Figure 1 the blue-colored solution obtained and showed an intense absorption band centered at *ca*. 669 nm with a weak shoulder at *ca*. 420 nm that can be assigned to the in-plane dipole and in-plane quadrupole plasmon resonances of AgNTs, respectively. The absence of bands around *ca*. 400 nm (typical of spheres) is indicative of the high yield of AgNTs obtained in this reaction (Millstone et al., 2009; Yu et al., 2014).

This colloid solution showed high sensitivity to the purification processes, using water as a dispersant. When the NPs were re-suspended in ultra-pure water, there was a fast blue shift of in-plane dipole plasmon band to *ca*. 566 nm and a disappearance of the in-plane quadrupole plasmon band, with a change of the color solution to purple (Figure 1). This behavior results from the enormous sensitivity of the AgNTs@PVP to suffer tip truncation, or partial dissolution during their manipulation, which strongly affects the final properties (Jiang et al., 2007).

Purification of the AgNTs@PVP using absolute EtOH as a dispersant did not produce this effect. As shown in Figure 1, the SPR band red-shifted from *ca*. 669 nm to *ca*. 701 nm as a consequence of the solvent change, which increases the dielectric constant of the medium (Link and El-Sayed, 1999; Szunerits and Boukherroub, 2012).

Another critical factor to be controlled was the centrifugation conditions during the purifications process. We observed that increases in the rotation speed, or in the time cycle, produced the formation of remarkable aggregates, especially when the AgNTs were resuspended in absolute EtOH. The selection of these purification conditions allowed us to obtain well-dispersed AgNTs in the EtOH solution. The final solution obtained was stable for several days without noticeable spectral changes.

**Figure 2:**
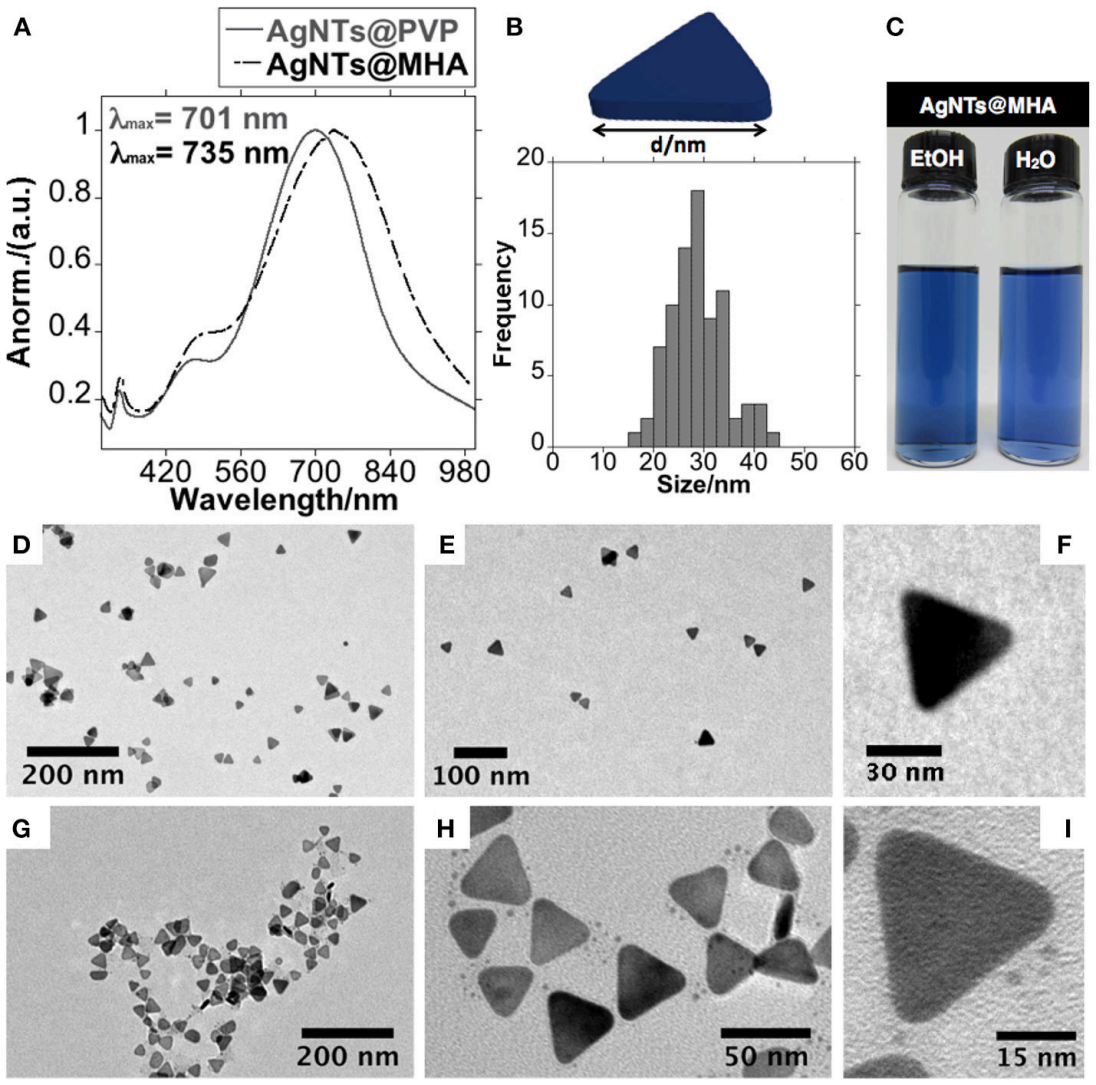
Spectroscopic profile of AgNTs@PVP and AgNTs@MHA in EtOH solution **(A)**, size histogram of lateral distance of AgNTs@MHA **(B)**, color solution of AgNTs@MHA resuspended in EtOH and water **(C)**. TEM images obtained of AgNTs@PVP **(D–F)** and AgNTs@MHA **(G–I)**.

Transmission electron microscopy (TEM) analysis of the colloid obtained in EtOH showed the AgNPs@PVP with triangular platelet geometry with an average size of 28.8 ± 5.4 nm and a disc width of 5.9 ± 0.9 nm (Figures 2B,D–F).

The marked sensitivity of AgNTs@PVP to manipulation in aqueous solution limits its potential application in antibacterial preparations.

Therefore, in order to increase the stability in aqueous solution, the organic functionalization of the AgNTs' surface was done by rapid addition of an ethanolic solution of 16-mercaptohexadecanoic acid (MHA) over the AgNTs@PVP solution under vigorous magnetic stirring, following previous published methods. The dipole plasmon resonant band red-shifted in 34 nm upon the organic functionalization with MHA (Xue et al., 2007) (Figures 2A,C).

As presented by the TEM images in Figures 2G–I, the anisotropic geometry does not show important structural modifications. The thiol-stabilized colloid presents high stability in both EtOH and water solution. The Z-potential analysis of AgNTs@MHA in aqueous solution showed a stable potential of −35.8 mV, confirming the presence of carboxylate groups on the surface of the AgNTs (Figure 5B).

**Figure 3:**
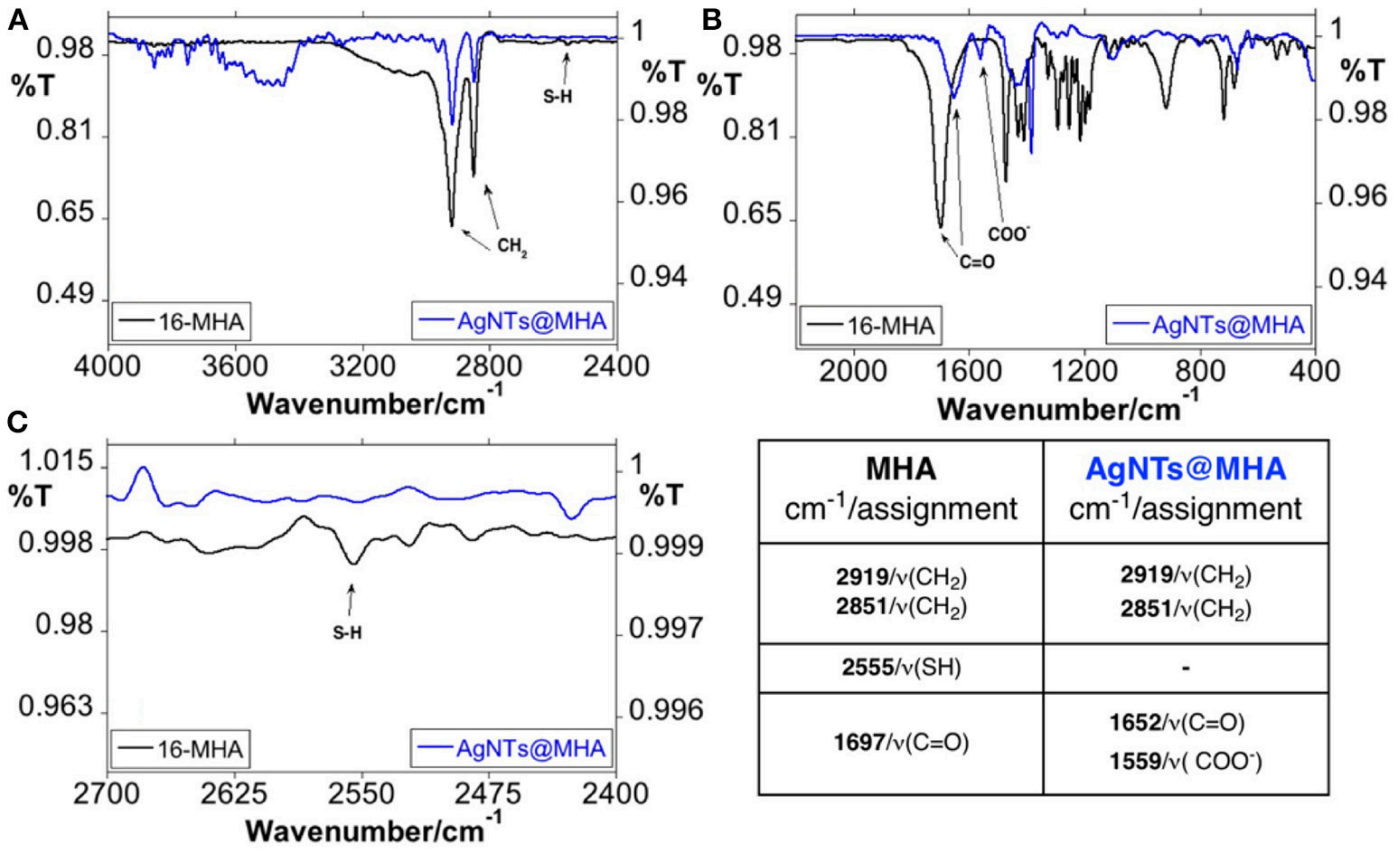
FT-IR spectroscopic profile of 16-MHA and AgNTs@MHA in KBr disk. Overview between 4,000 and 2,400 cm^−1^ **(A)** and 2,000–400 cm^−1^ **(B)**. Enlargement spectra in the S-H region between 2,700 and 2,400 cm^−1^**(C)** and peak table.

The FT-IR spectroscopy was employed to inspect the composition of this colloid solution. The CH_2_ stretching vibrations peaks detected in AgNTs@MHA correspond to the frequencies observed in the free MHA at 2,919 and 2,851 cm^−1^ (Morales-Cruz et al., 2005). Contrary to the spectrum of the pure compound, for AgNTs@MHA no signals were detected at 2,555 cm^−1^, which was assigned to the ν(S-H) stretching vibration. This fact is indicative, on the one hand, that MHA molecules have adsorbed to the AgNTs' surface through the sulfur group, and on the other hand, of the absence of unreacted MHA molecules on the AgNTs@MHA colloid suspension (Johnson et al., 1998; Morales-Cruz et al., 2005; Gupta et al., 2012). The band observed at 1,559 cm^−1^ in AgNTs@MHA could be assigned to ν(COO^−^) symmetric stretch (Morales-Cruz et al., 2005) as a consequence of a partial ionization of the carboxylic group of MHA molecules exposed at the silver surface. Finally, the signal observed at 1,652 cm^−1^ could be related to the ν(C=O) stretching vibration of carboxylic acid in MHA, or also to the ν(C=O) stretching vibration amide group of remnant PVP adsorbed on the surface of AgNTs@MHA (Figure 3).

These organic coating AgNTs (AgNTs@MHA) were selected for the antibacterial studies.

### Silica Coating of AgNTs@MHA

As reported previously by Mirkin et al. thiol-stabilized AgNTs can be used satisfactorily in the next control of the coating with silica, without affecting the anisotropic structure of the AgNTs (Xue et al., 2007). In that work, the authors started with silver nanoprisms synthesized through a photochemical process, using a single beam excitation system in the presence of citrate and bis (p-sulfonatophenyl) phenylphosphine (BSPP). Then, the AgNTs were functionalized with MHA, and later with a thin and highly uniform silica coating shell using TEOS as a precursor of silica, and DMA as a catalyst.

In our case, we used AgNTs@PVP as starting materials to obtain AgNTs@MHA, and then to produce AgNTs@Si. The silica coating offers an exciting possibility to study how the antibacterial properties of AgNTs@MHA are affected when they are subjected to a dense inorganic coating.

Our intention concerning the silica deposition was to explore the synthetic conditions that allow obtaining a thin but uniform coating, without substantially affecting the anisotropy of the nanoparticles. In this way, keeping constant the DMA concentration (0.5 M) and the reaction time (180 min), we have explored the coating process obtained using different TEOS concentrations between 0.9 and 0.5 mM. As can be seen in Figure S1, in our case, the decrease in the concentration of TEOS during the coating does not permit obtaining a fine homogeneous silica coating. Additionally, all the analyzed samples showed in Figure S1 presented an essential loss of the triangular geometry of the platelets. Interestingly, for the higher concentrations of TEOS explored, we detected the presence of holes within the silica nanostructure (Figure S1a).

Considering the nanostructures obtained, the dissolution process suffered by AgNTs during the silica deposition seems to be affected to a large extent by the contact time of the nanostructures with the DMA and/or the concentration of the same.

Based on the previous observations related to the TEOS concentration, using 0.5 mM, we have decreased the reaction time to 90 min. In Figure S2, it can be seen after just 90 min. of reaction, the silica coating showed a homogenous character overall of silver cores. Remarkably, we conclude that in our process, the re-shaping of AgNTs is preserved to a greater extent for the reaction obtained with 90 min, indicating that more significant contact with DMA produces higher re-shaping of the AgNTs, probably by diffusing of DMA through the already-formed silica shell.

**Figure 4:**
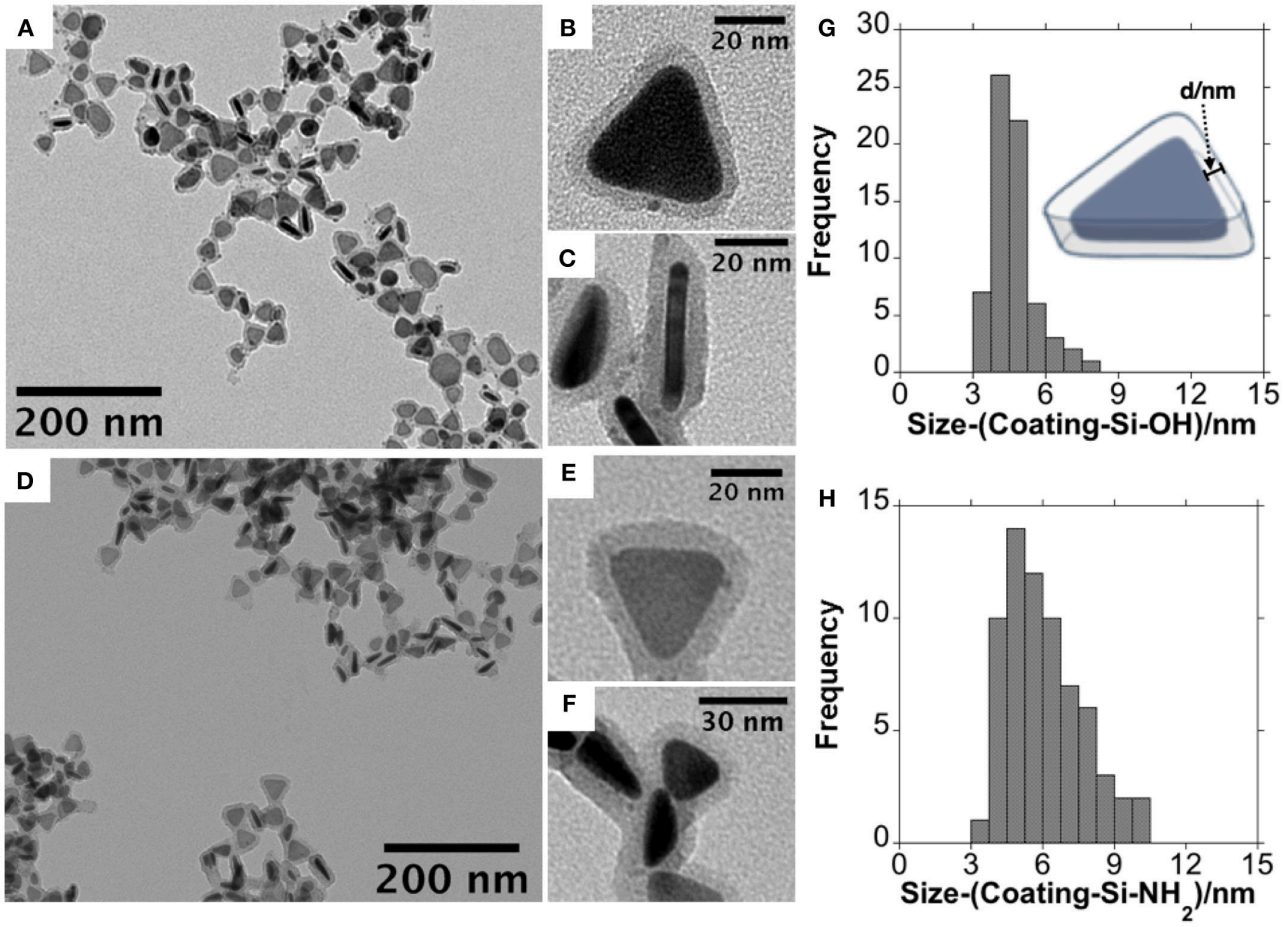
Low magnification TEM images at different magnifications obtained for AgNTs@Si-OH **(A–C)** and AgNTs@Si-NH_2_ **(D–F)** and size histogram of silica coating for AgNTs@Si-OH **(G)** and AgNTs@Si-NH_2_ **(H)**.

Finally, for a concentration of TEOS of 0.5 mM, and 90 min of reaction, we have explored the decrease in the concentration of DMA to 0.4 M. The reduction of the concentration of DMA not only allowed us to preserve to a greater extent the anisotropy of the AgNTs, but also allowed us to obtain a relatively homogeneous silica coating. Consequently, the refined synthetic conditions were [TEOS] = 0.5 mM, [DMA] = 0.4 M and 90 min., allowing us to obtain a silica coating thickness of around 5 nm (Figures 4A–C,G).

**Figure 5:**
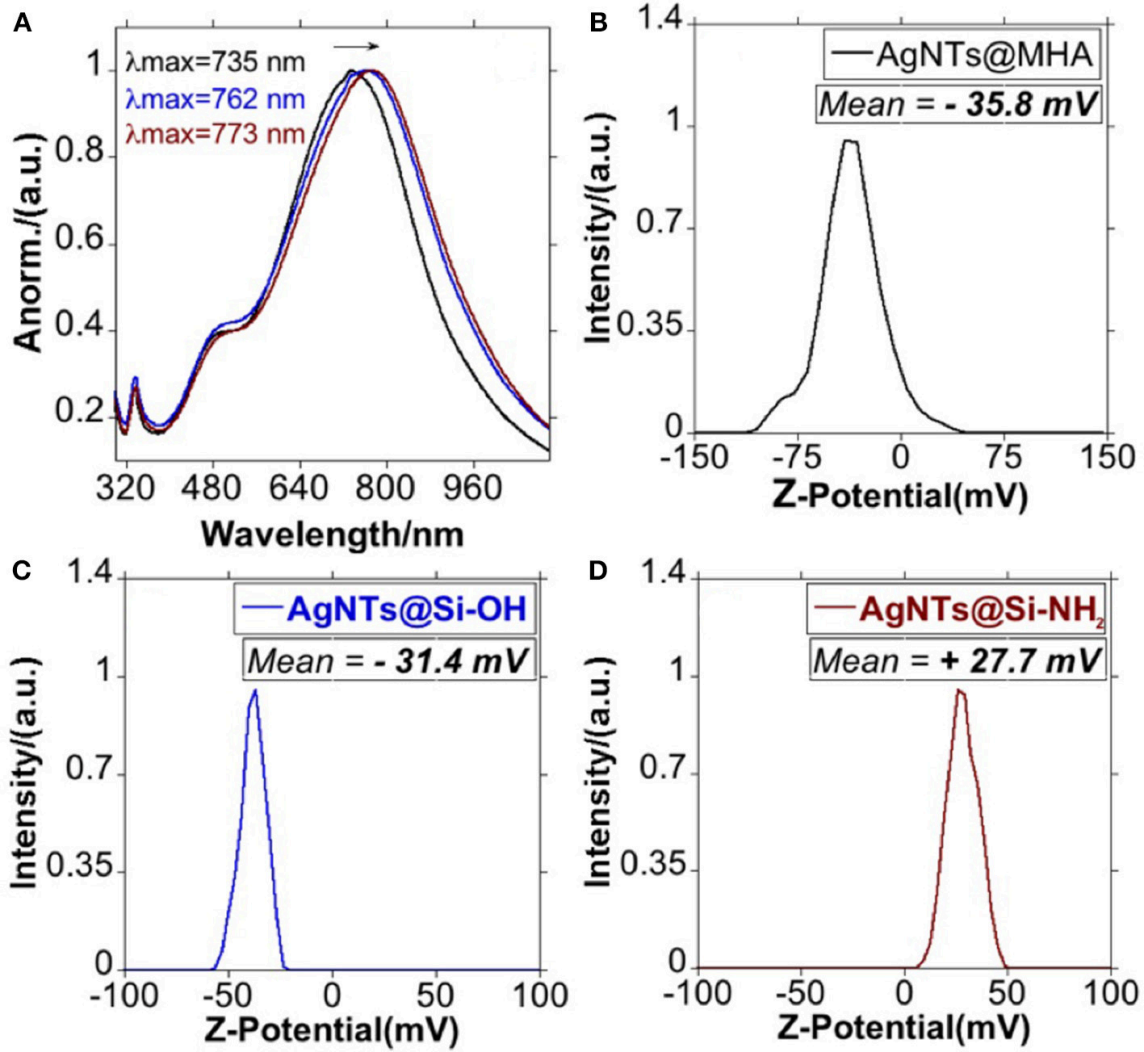
Spectroscopic profile of AgNTs@MHA, AgNTs@Si-OH and AgNTs@Si-NH_2_ in EtOH **(A)**, and graphic representation of the Z-potential for AgNTs@MHA **(B)**, AgNTs@Si-OH **(C)**, and AgNTs@Si-NH_2_ **(D)**.

The dipole plasmon resonant band of AgNTs@MHA red-shifted in 27 nm, upon silica coating (Figure 5A), following previous similar reports (Xue et al., 2007).

**Figure 6:**
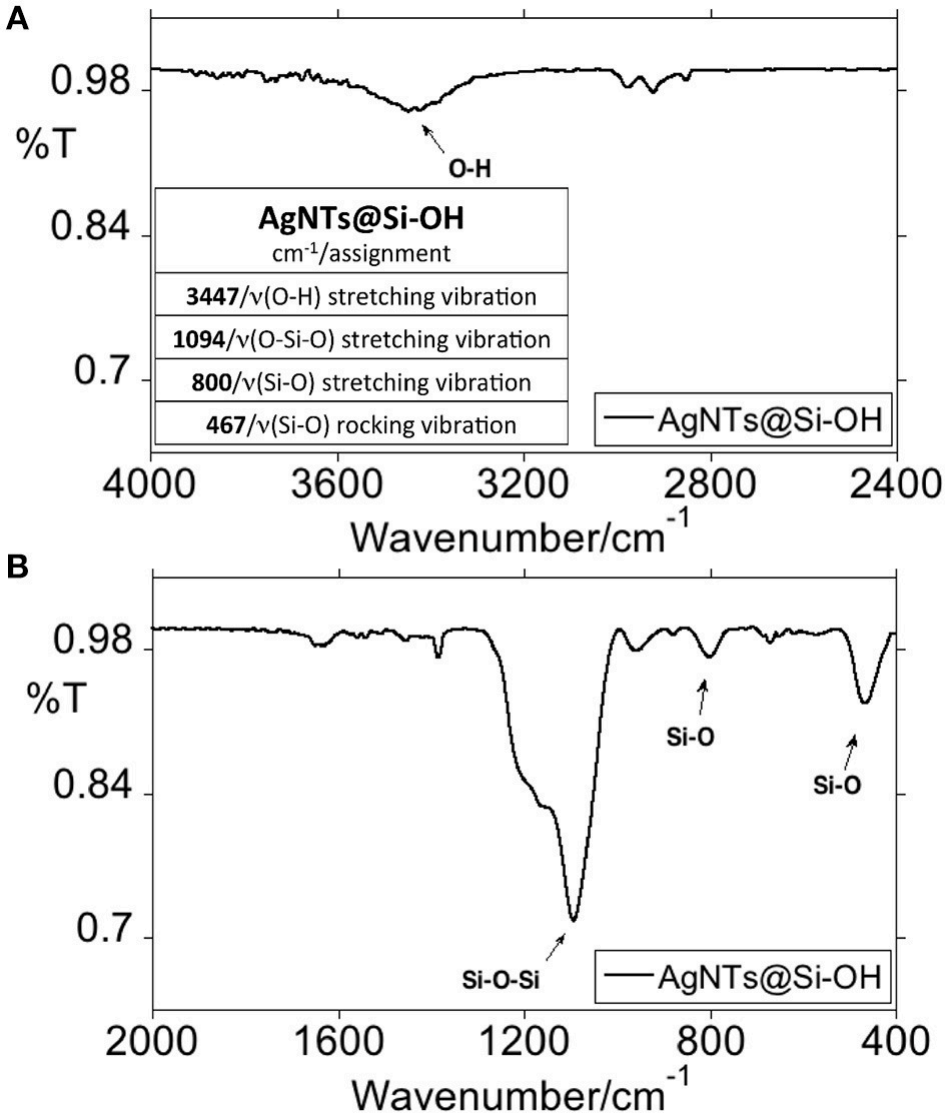
FT-IR spectroscopic profile of AgNTs@Si-OH in KBr disk. Overview between 4,000 and 2,400 cm^−1^ and peak table **(A)** and 2,000 and 400 cm^−1^**(B)**.

Using FT-IR spectroscopy, the characteristic signals for the silica were observed at 467, 800, 960, and 1,094 cm^−1^ (Figure 6). These signals can be assigned to the bending vibration of Si-O-Si, stretching, and bending vibrations of Si-OH, and asymmetric stretching vibration of O-Si-O, respectively (Rahman et al., 2009; Azarshin et al., 2017; Sakthisabarimoorthi et al., 2017), confirming the polymerization of the silane on the silver cores.

In a subsequent step, we have derivatized the terminal -OH group of the AgNTs@Si-OH into –NH_2_ through a silane coupling reaction with APTMS, based on established protocols (Bruce and Sen, 2005). As shown by the TEM images in Figures 4D–F through APTMS coupling on AgNTs@Si-OH, we obtained a slight increase in the shell silica size (≈1.3 nm) (see Figure 4H). This functional group conversion can be readily confirmed using Z-potential analysis of the colloids in water. Therefore, the AgNTs@Si-OH have a surface charge equal to −31.4 mV, which is reversed to positive values +27.7 mV for AgNTs@Si-NH_2_ (Figures 5C,D). This Zeta potential value reversion is the consequence of the different energies of ionization in water presented by the -OH and -NH_2_ groups (Jacobasch, 1989; Jesionowski, 2003).

These positively-charged **AgNTs@Si-NH**_**2**_ were selected as the second sample for bacteriological analysis.

**Figure 7:**
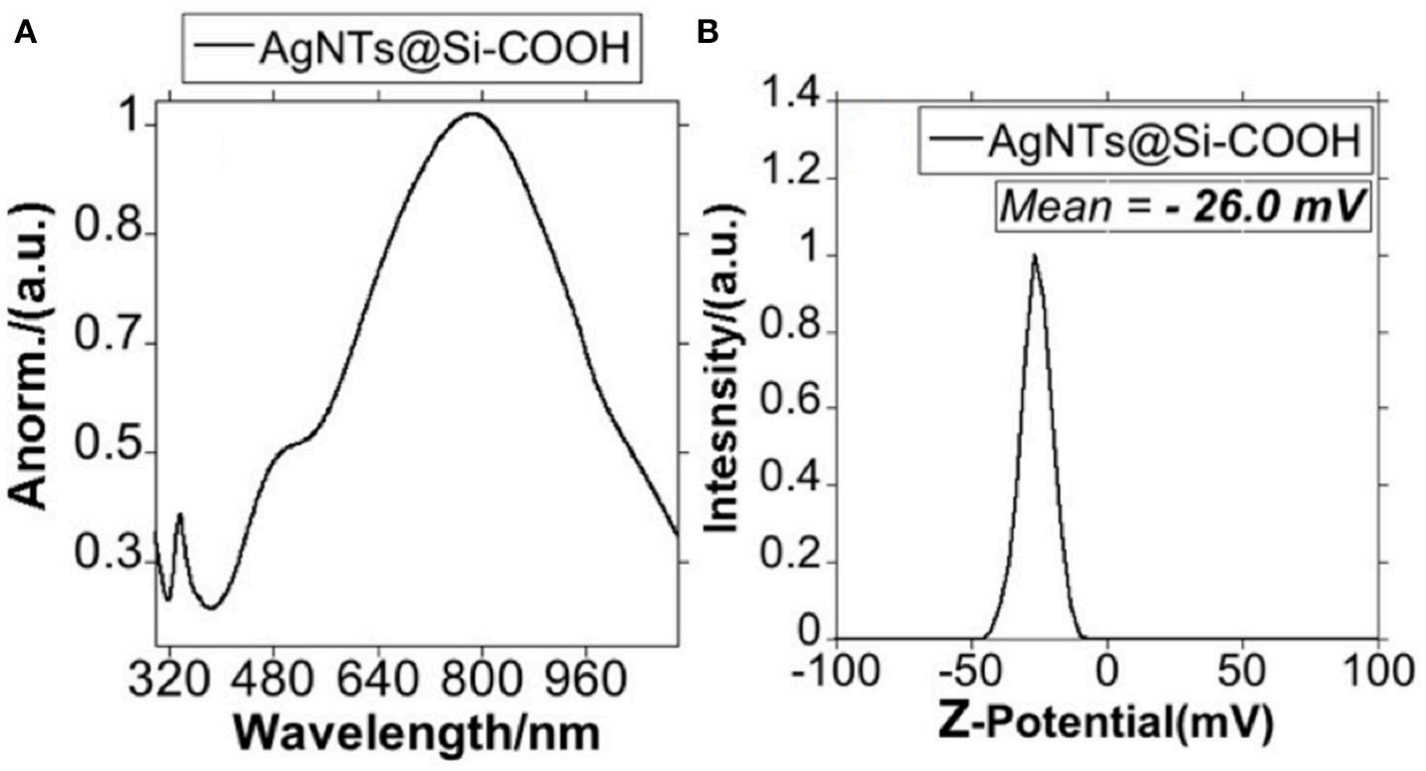
Spectroscopic profile of AgNTs@Si-COOH **(A)** and graphic representation of the Z-potential for AgNTs@Si-COOH **(B)**.

Finally, to explore if the effect of surface charge can affect the antimicrobial properties of silica-coated AgNTs, we converted the terminal organic amine group (-NH_2_) into carboxylic acid (-COOH), reacting AgNTs@NH_2_ with succinic anhydride in anhydrous THF. As can be seen in Figure 7A, the LSRP was not significantly affected. On the other hand, upon conversion of an amine into the carboxylic group, the Z-potential of the colloid in ultrapure water moved from +27.7 mV to −26.0 mV, which was in accordance with previous reports about AgNPs@Si functionalization (Bahadur et al., 2011) (Figure 7B).

The negatively-charged **AgNTs@Si-COOH** were selected as the third sample for bacteriological analysis.

### Exploring Bactericidal Properties

The antibacterial susceptibility tests were performed by the broth dilution method. Previous studies have proved that silver nanoparticles alone and silver composites have high antibacterial effectiveness against bacteria, fungi and viruses (Akhavan and Ghaderi, 2009; Liga et al., 2011; Ifuku et al., 2015) (see Figure S1 and Figure S3). The minimum inhibitory concentration (MIC) and the minimal bactericidal concentration (MBC) values are shown in Table 2.

**Table 2:** The minimum inhibitory concentration (MIC) and minimum bactericidal concentration (MBC) of AgNTs@MHA, AgNTs@Si-NH_2_, and AgNTs@Si-COOH toward *E. coli* K12 ATCC 29425 and *S. aureus* ATCC 25923.

**Sample**	**Strain**	**MIC (μg/ml)**	**MBC (μg/ml)**
AgNTs@MHA	*E. coli* K12 ATCC 29425	10	10
	*S. aureus* ATCC 25923	5	10
AgNTs@Si-NH_2_	*E. coli* K12 ATCC 29425	25	50
	*S. aureus* ATCC 25923	5	10
AgNTs@Si-COOH	*E. coli* K12 ATCC 29425	25	50
	*S. aureus* ATCC 25923	10	25

Based on the MIC/MBC values obtained for the three selected samples, we have confirmed that the AgNTs that were only subjected to organic coating (AgNTs@MHA) showed the best antimicrobial properties, with MIC/MBC values of 10/10 and 5/10 (μg/mL) for *E. coli* and *S. aureus*, respectively. Compared with other studies using different spherical silver NPs, AgNTs@MHA show suitable antimicrobial properties.

For instance, the AgNPs@citrate ranged in size between 5 and 100 nm, showing MICs that varied from 20 to 160 μg/mL for the two tested strains of *E. coli* (Agnihotri et al., 2014). Besides, AgNPs@PVP in a range of sizes between 9 and 16 nm showed higher MBC values than those obtained in our case for AgNTs@MHA for the same bacterial strain *S. aureus* (ATCC 25923) (Bryaskova et al., 2011).

Note that in our previous study using 15 nm spherical AgNPs stabilized with tetracycline, higher MIC values against the same bacterial strains studied in the present work were visible (between 16 and 32 μg/mL; Djafari et al., 2016). These important results show how the rational selection of the shape in silver NPs could overcome the synergistic effect produced by spherical shape and tetracycline.

The present results, therefore, are in agreement with previous works which elucidate a greater antibacterial effect of nanoparticles with {111} basal plane (Morones et al., 2005). More specifically, Sadeghi and co-workers studied the antimicrobial effect of PVP-stabilized nanospheres, nanobars, and nanoplates against *E. coli* and *S. aureus* (Sadeghi et al., 2012). These authors proved a marked increase in the antimicrobial effect for nanoplates when compared to nanobars and nanospheres, as a consequence of higher surface area observed for nanoplates.

Studies on the impact of the AgNTs' surface coating on antibacterial activity remain limited to organic coating, and have highlighted antibacterial properties (Tang et al., 2013; Marta et al., 2014; Lu et al., 2015; D'Agostino et al., 2017; Tanvir et al., 2017). For instance, pluronic-coated nanoprisms have been successfully used as a bactericidal agent against two methicillin-resistant *S. aureus* strains. These authors observed strong bacteriostatic and bactericidal activity related to the high sensitivity of the tips and edges of AgNTs to undergo oxidation (Marta et al., 2014).

However, the inorganic coating of AgNTs has never been explored for antibacterial applications. Silica is presented as an ideal candidate since it favors stability to oxidation (Brandon et al., 2014), decreases non-specific interactions between the metallic surface and biomolecules (Bagwe et al., 2006), increases solubility in aqueous media or facile production and post-functionalization processes (Bahadur et al., 2011), among others advantages.

Related the samples subjected to silica coating, a decrease in the antibacterial effect concerning AgNTs@MHA can be noted by the MIC/MBC values summarized in Table 2. We believe that the subsequent coating of AgNTs@MHA with silica should delay the dissolution processes of AgNTs, decreasing the Ag^+^ ratio released.

Despite this, the MIC/MBC values obtained between 5 and 50 μL/mL showed notably antimicrobial effects. More important, the bactericidal effect against *S. aureus* ATCC 25923 was not altered after silica coating for the case of AgNTs@Si-NH_2_, indicating that the release of silver ions is probably not a determinant factor in the antibacterial activity of positively charged AgNTs against this specific strain. Note that the antibacterial effect obtained for AgNTs@Si-COOH in the two bacterial strains studied was decreased when compared to AgNTs@MHA. Therefore, the surface charge of AgNTs should be considered in the mode of action against *S. aureus*. In this regard, it has been previously suggested that positively-charged AgNPs grant a higher antimicrobial effect when compared to similar negatively-charged NPs (Abbaszadegan et al., 2015).

Finally, and supporting our results, it has been pointed out by different authors that after the silica coating the silver nanoparticles, AgNPs retain their antibacterial properties (Xu et al., 2009; Le et al., 2010).

## Conclusions

We have successfully synthesized silver nanotriangles, AgNTs@PVP, in aqueous solution. Afterward, functionalization with 16-mercaptohexadecanoic acid (AgNTs@MHA) and the subsequent silica deposition were deeply investigated. We have determined the ideal synthetic conditions to obtain amorphous silica coating with ≈5 nm. Terminal amine functionality was introduced through a chemical reaction with APTMS, increasing the shell thickness to ≈1.3 nm. Amino group terminated nanoparticles (AgNTs@SiNH_2_) then reacted with succinic anhydride in THF to obtain AgNTs@Si-COOH. The bacterial properties of AgNTs with a molecular coating (AgNTs@MHA) and silica coating (AgNTs@Si-NH_2_ and AgNTs@Si-COOH) were investigated against Gram-positive and Gram-negative bacteria.

Comparing the three explored samples, the higher antibacterial effect was observed for the silica-free sample as expected, showing the best MIC values against *S. aureus* (ATCC 25923)—equal to 5 μg/mL. We have observed that the silica coating decreases the antibacterial effect for all the strains studied, except for the case of the positively-charged AgNTs@Si-NH_2_ against *S. aureus* (ATCC 25923). These results indicate that the release of silver ion is not the unique critical point in the mode of action of AgNTs; nonetheless, the surface charge must also be taken into consideration. Even so, the MIC/MBC values for the silica-coated samples showed a similar range to the values reported in the literature for another type of uncoated-silica AgNPs.

As an important final remark, it should be mentioned that the high aqueous colloidal stability and the presence of terminal organic groups (COOH or NH_2_) in the explored nanomaterials, both open the door to the design of more sophisticated nano-antibiotics through rational organic functionalization with bactericide-active molecular agents. Currently the application of these materials as building blocks to produce hybrid nano-antibiotics is under development in our laboratory.

## Author Contributions

JF-L, JLC and CL designed and supervised the project. JD, CF-L, AF-L, and JF-L performed the synthetic experiments. JF-L, CL, JLC, GI, JD, AF-L, and CF-L analyzed the results. JF-L and CL wrote the first draft. CL, JF-L, JLC, and GI provided the resources related to the project. JF-L, JD, CF-L, and AF-L produced all graphical materials. All authors reviewed and corrected the final manuscript. CL, JLC, and GI financed the project. PP and GI designed the bactericidal experiments. VS performed the bactericidal experiments.

### Conflict of Interest Statement

The authors declare that the research was conducted in the absence of any commercial or financial relationships that could be construed as a potential conflict of interest.
