# Supplementary material for: Exploring the Control in Antibacterial Activity of Silver Triangular Nanoplates by Surface Coating Modulation
Source: Front Chem. 2019 Feb 5;6:677. doi: 10.3389/fchem.2018.00677 (PMC6370693; doi:10.3389/fchem.2018.00677)
Supplement: Supplementary file 1 [file Data_Sheet_1.docx]

**Exploring the control in antibacterial activity of silver triangular nanoplates by surface coating modulation**

Jamila Djafari,^1,2,3^ Carlos Fernández-Lodeiro,^1,2^ Adrián Fernández-Lodeiro,^1,2,3^ Vanessa Silva,^3-6^ Patrícia Poeta,^3,6^ Gilberto Igrejas,^3,4,5^ Carlos Lodeiro,^1,2,3^ José Luis Capelo,*^1,2,3^, Javier Fernández-Lodeiro,*^1,2,3^

^1^ BIOSCOPE Group, LAQV@REQUIMTE, Chemistry Department, Faculty of Science and Technology, NOVA University Lisbon. Caparica Campus. 2829-516 Caparica. Portugal;

^2^ PROTEOMASS Scientific Society, Rua dos Inventores, Madam Parque, Caparica Campus, 2829-516 Caparica, Portugal;

^3^ Associated Laboratory for Green Chemistry (LAQV-REQUIMTE), University NOVA of Lisbon, Caparica, Portugal;

^4^ Department of Genetics and Biotechnology, University of Trás-os-Montes and Alto Douro, Vila Real, Portugal;

^5^ Functional Genomics and Proteomics Unit, University of Trás-os-Montes and Alto Douro, Vila Real, Portugal;

^6^ Veterinary Science Department, University of Trás-os-Montes and Alto Douro, Vila Real, Portugal;

*** Correspondence:**Javier Fernández-Lodeiro (j.lodeiro@fct.unl.pt)/ José Luís Capelo (jlcm@fct.unl.pt)

Keywords: Silver triangular nanoplates, silica coating, succinic anhydride, APTMS, antibacterial properties

1. Additional characterizations

***
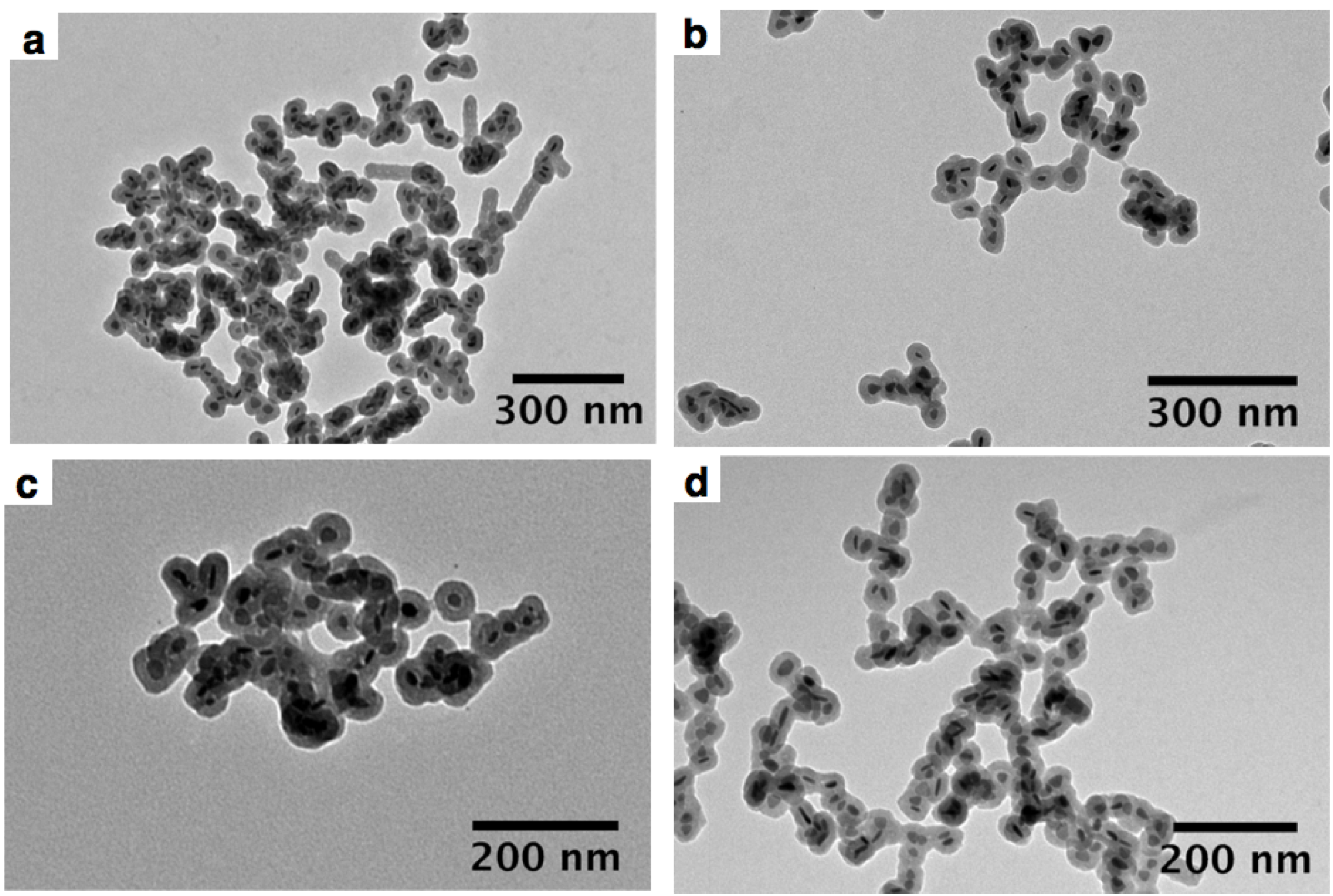
***

***Figure S1****: Low-resolution transmission electron microscopy (TEM) images in different magnification of AgNTs@Si-OH obtained under [DMA]=0.5 M and 3 hours of reaction using different [TEOS], 0.9mM (****a****), 0.7 mM (****b****), 0.6mM (****c****) and 0.5 mM (****d****).*

***
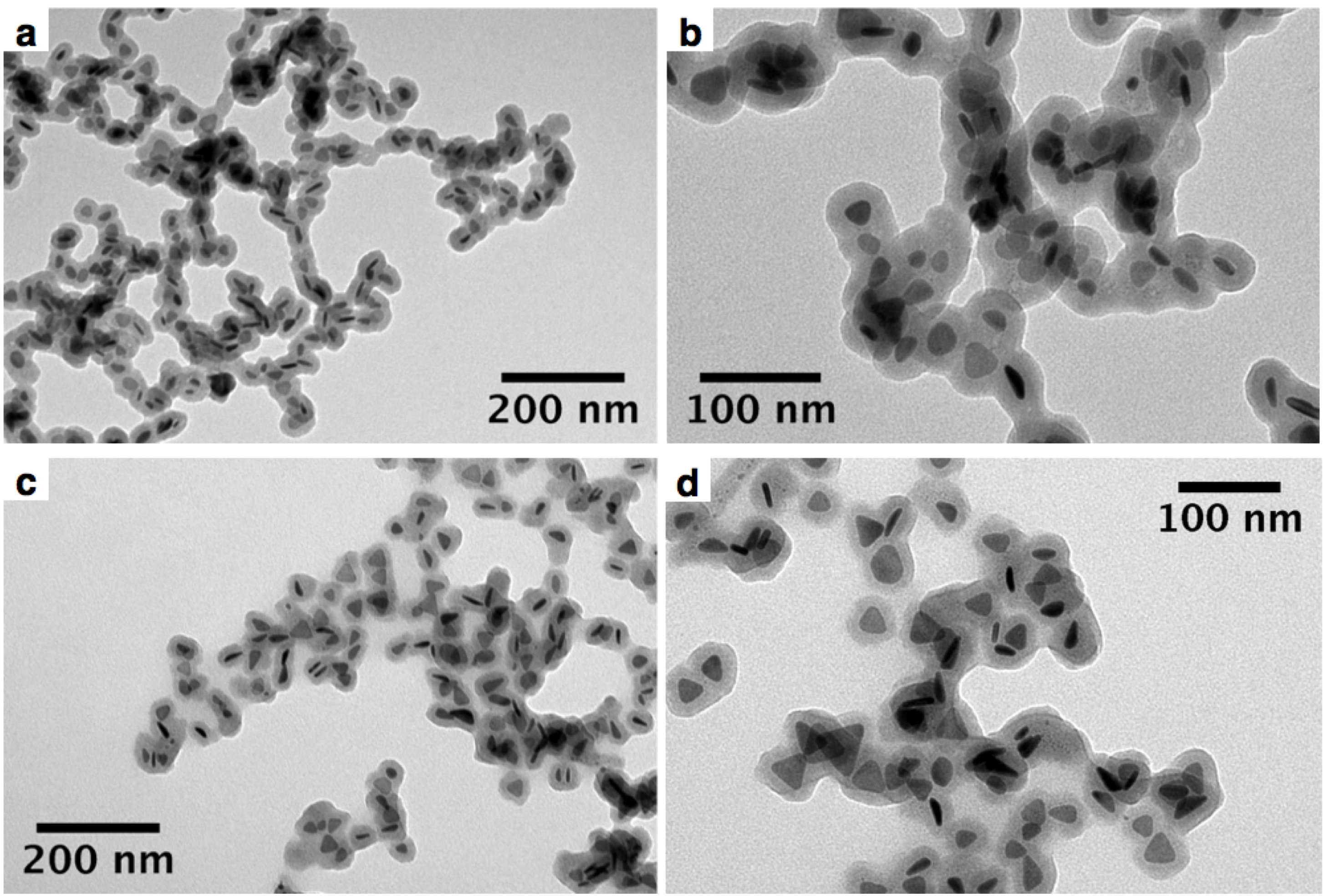
***

***Figure S2:*** *Low-resolution transmission electron microscopy (TEM) images in different magnification of AgNTs@Si-OH obtained under [TEOS]=0.5mM, [DMA]=0.5 M for 180 min (****a and b****) and* 90 min.  *(****c and d****).*

***2. Proposed antibacterial mechanism***

The antibacterial effect of silver ions and AgNPs has been known for decades. Silver ions (Ag^+^) are reported to disrupt the outer membrane of target bacterial cells, however, this effect has been observed at concentrations much more higher than those used for AgNPs. The antibacterial effect of AgNPs may be due to the large surface area provided by the AgNPs which facilitates the attachment of the nanoparticles to the cell membrane and, consequently, an easier penetration into the bacteria (Wong and Liu, 2010). Several studies and reviews (Durán et al., 2016; Mijnendonckx et al., 2013; Reidy et al., 2013) have elucidated the probable modes of action of AgNPs: 1) the AgNPs adhere and accumulate on the bacteria surface then penetrating the cell wall which can cause structural changes in the cell membrane leading to a higher permeability. Besides, the accumulation of AgNPs on the bacterial membrane produces gaps compromising the integrity of the membrane causing increased permeability and, consequently, cell death; 2) the AgNPs may release silver ions that interact with the enzyme’s thiol groups inactivating them. The silver ions may also enter the cell membrane inhibiting some cell functions; 3) silver ions may inhibit the respiratory chain enzymes or interfere with membrane permeability due to the uncoupling of respiratory electron transport from oxidative phosphorylation resulting in impaired DNA replication. The reactive oxygen species (ROS) are produced through the inhibition of respiratory chain enzymes which have a known powerful antibacterial activity; 4) the silver ions have the ability to enter the bacterial cell, inhibiting the protein synthesis and interfering with the translation and transcription; 5) finally, silver is an acid and the cells are mostly made out basic components (phosphorous and sulfur), therefore, they tend to react with each other resulting in cell death. Besides, the major components of DNA are also bases, and the AgNPs may interfere with them destroying the DNA of the bacterial cell.

We believe that the mechanism of action of AgNTs should proceed in a similar way to that observed for spherical nanoparticles, but with increased Ag^+^ release ratios, as well as a greater surface adsorption on bacteria. **Figure S3** illustrates the possible mechanisms of the antibacterial effects of AgNTs.

**
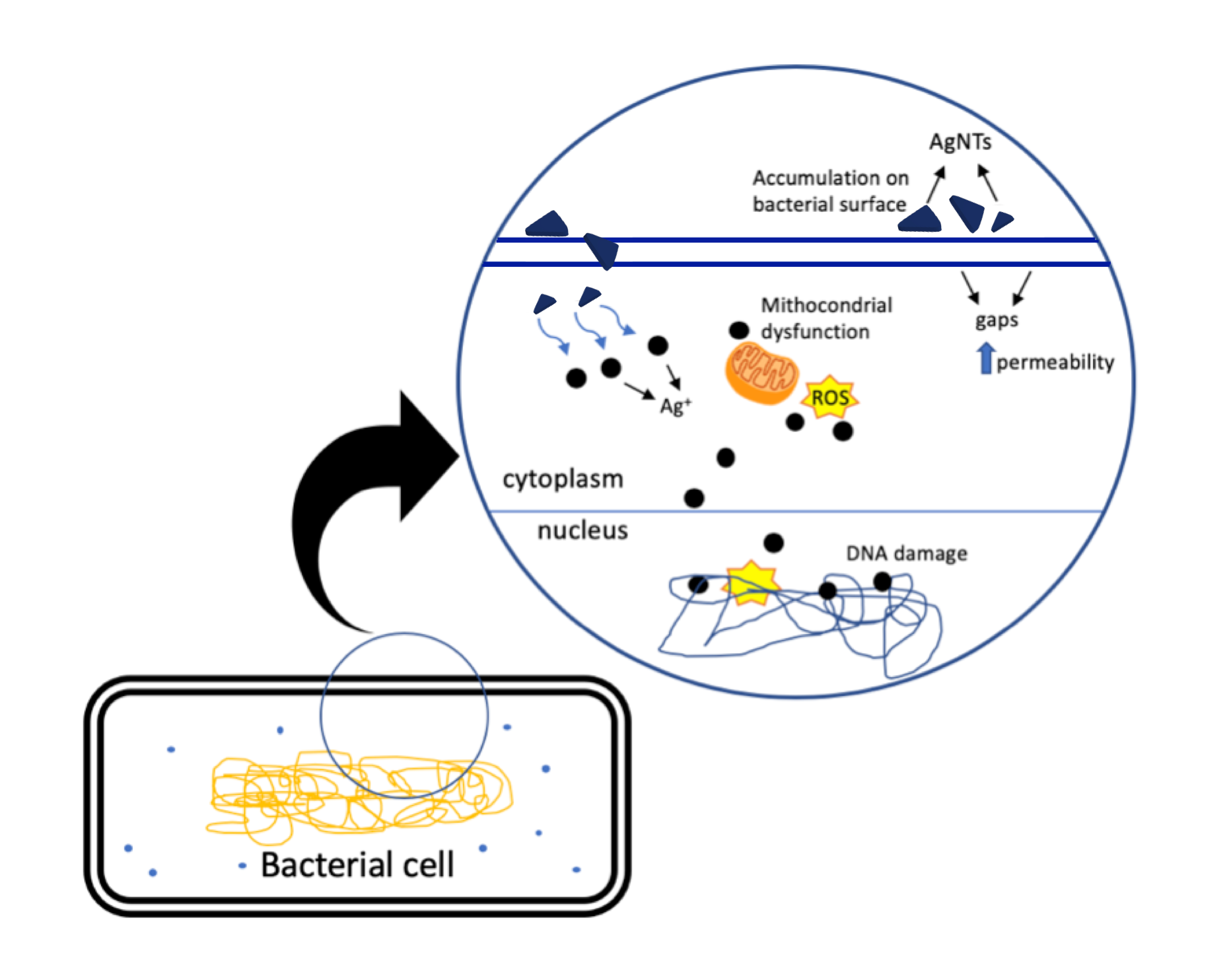
**

*Figure S3:* – Plausible antibacterial mechanisms of AgNTs.

***3. References***

Durán, N., Durán, M., de Jesus, M. B., Seabra, A. B., Fávaro, W. J., and Nakazato, G. (2016). Silver nanoparticles: A new view on mechanistic aspects on antimicrobial activity. *Nanomedicine Nanotechnology, Biol. Med.* 12, 789–799. doi:10.1016/j.nano.2015.11.016.

Mijnendonckx, K., Leys, N., Mahillon, J., Silver, S., and Van Houdt, R. (2013). Antimicrobial silver: uses, toxicity and potential for resistance. *BioMetals* 26, 609–621. doi:10.1007/s10534-013-9645-z.

Reidy, B., Haase, A., Luch, A., Dawson, K., and Lynch, I. (2013). Mechanisms of Silver Nanoparticle Release, Transformation and Toxicity: A Critical Review of Current Knowledge and Recommendations for Future Studies and Applications. *Materials (Basel).* 6, 2295–2350. doi:10.3390/ma6062295.

Wong, K. K. Y., and Liu, X. (2010). Silver nanoparticles—the real “silver bullet” in clinical medicine? *Medchemcomm* 1, 125. doi:10.1039/c0md00069h.
